# Differences in dynamic perception of salty taste intensity between young and older adults

**DOI:** 10.1038/s41598-022-11442-y

**Published:** 2022-05-09

**Authors:** Hitomi Sato, Hirotaka Wada, Hideki Matsumoto, Mutsumi Takagiwa, Tazuko K. Goto

**Affiliations:** 1 Department of Oral and Maxillofacial Radiology, Tokyo Dental College, 2-9-18 Kandamisaki-cho, Chiyoda-ku, Tokyo, 101-0061 Japan; 2 Tokyo Dental College Research Branding Project, Tokyo Dental College, 2-9-18, Kanda-Misakicho, Chiyoda-ku, Tokyo, 101-0061 Japan; 3 Laboratory of Mathematics, Tokyo Dental College, 2-9-18 Kandamisaki-cho, Chiyoda-ku, Tokyo, 101-0061 Japan; 4 Faculty of Dentistry, The University of Hong Kong, 34 Hospital Road, Sai Ying Pun, Hong Kong, China

**Keywords:** Neuroscience, Health care

## Abstract

In super-aged societies, high salt intake substantially increases the risk of stroke and cardiovascular disease. Perceived low salty taste often prompts the addition of table salt to food. However, it remains unclear how older adults perceive the nature and intensity of salty taste in the mouth and brain. We compared the perceptions of salty taste intensities of older adults with those of young adults. Participants were 74 healthy adults: 31 older (age, 60–81 years [65.0 ± 5.5 SD]) and 43 young (age, 21–39 years [25.0 ± 3.6 SD]). Our research project comprises three sequential experiments. This article reports on the first two, which were (1) static and (2) dynamic sensory evaluations of taste perceptions in the mouth. Participants assessed the taste of 0.3 M and 0.5 M sodium chloride solutions in two types of sensory evaluations: (1) a cup tasting test, in which they sipped the solution from cups, spat it out, and rated static salty taste intensity, and (2) a time-intensity sensory evaluation, in which the solutions were delivered to participants’ tongues through a custom-made delivery system while they recorded dynamic taste intensities on a hand-held meter. Older adults perceived significantly lower taste intensities than young adults (*p* = 0.004 and *p* < 0.001 for 0.3 M and 0.5 M, respectively). Reaction timings for both solutions did not differ, but the slopes for both concentrations were significantly lower for older adults than for young adults (*p* < 0.001). Using a standardized system allowed us to evaluate and directly compare real-time feedback on taste intensities according to age. This study is the first to characterize the time-intensity profiles of salty taste intensity in older adults. Our findings show that older adults do not take longer to recognize a salty taste, but their perception of taste intensity slowly increases, and yet remains lower than that of young adults. This suggests that older adults should be aware of the tendency to add more salt to their food to compensate for their low perceptions of salty taste. We would like to suggest them to savor and chew sufficiently during eating to optimize the perceived salty taste. Furthermore, our results offer a reference for ordinary citizens’ taste-intensity perceptions; our standardized system could be usefully integrated into clinical follow-up examinations and treatments.

## Introduction

Taste is very important for both nutritional evaluation of food and for quality of life. In fact, a decline in taste sensitivity may reduce the pleasure associated with eating meals and exacerbate poor nutritional status. Some reports suggest that aging causes a decline in taste sensitivity^1–3^. To compensate for the reduction in salty taste perception, older adults often increase their salt intake. According to the National Health and Nutrition Survey in Japan, salt intake for both men and women is higher in people aged 60–70 + years than in people aged 20–29 and 30–39 years^4^. High salt intake substantially increases the risk of stroke and cardiovascular disease^5–8^.

The extent of age-related decline in taste varies for different tastes (e.g., salty, sweet, sour, bitter, and umami)^9–11^. A decline in taste sensitivity in older adults can result from normal aging, but mainly stems from certain disease states, medications, surgical intervention, and environmental exposure. The mechanisms underlying taste changes in normal aging (i.e., without considering the effects of disease and medication) are not fully understood^12^.

To understand how older adults perceive the nature and intensity of salty taste, we assumed both the mouth and brain should be investigated. Therefore, our study consisted of the following three sequential experiments.

- Experiment (1) cup tasting test (static sensory evaluation).
- Experiment (2) time-intensity sensory evaluation (dynamic sensory evaluation).
- Experiment (3) functional magnetic resonance imaging (fMRI)(future study: functional brain imaging to elucidate brain activations by taste).

In this paper, we focus on taste perception in the mouth, and thus report only on experiments (1) and (2), because they have provided significant important results for taste perception research. Additionally, a detailed data analysis of (1) and (2) are essential for experiment (3) because the results will allow us to: (i) investigate the correlation between perception in the mouth and in the brain, and (ii) more rigorously design the fMRI research (e.g., how many seconds we will deliver water to wash out the taste solution on the tongue during fMRI scanning).

Taste perception may be related to anatomical changes in the taste buds. Taste bud density and taste bud cell density of the circumvallate papillae in human cadavers are lower in adults older than 76 years than in individuals aged 0–15 years^13^. Living individuals older than 60 years show a fungiform papillae morphology of substantial deterioration and a substantially reduced density, along with an increase in detection thresholds for the ‘electric taste’^14^. However, the number of taste buds and fungiform papillae are not substantially associated with age in cadavers^15,16^. Findings from anatomical studies on age-related changes in taste buds are equivocal.

Several different types of sensory taste evaluations have been used. One experimental method seeks to determine detection thresholds (the minimum concentration at which participants can reliably discriminate the taste from water) and recognition thresholds (the minimum concentration at which participants can identify the taste quality, such as salty for sodium chloride, NaCl). Another method assesses the suprathreshold (the sufficient strength at which taste quality is perceived in daily life).

Substantial age-associated taste deterioration has been reported based on detection thresholds and recognition thresholds using a whole-mouth method^17^, a filter disk test^1^, or a taste-strip method^18^. Some researchers have found no differences between suprathreshold responses to NaCl and sucrose in young and older adults^19–21^; conversely, others have reported that the intensity rating of suprathreshold tastes declines with age^22–25^.

In this study, we focused on age-related decline in taste for solutions with suprathreshold intensities. Previous findings on suprathreshold levels vary because the evaluation methods differ across studies. To obtain data under standardized conditions, we used the oral device and taste delivery system developed by Goto et al.^26^. This permitted us to obtain sensory evaluation data on taste-intensity changes over time and examine the differences between　older and young adults. The standardized conditions are described in the ‘Time-intensity sensory evaluation’ section in the “Materials and Methods” and the “Discussion”. This system delivers a constant amount of taste solution to the whole tongue. Participants experienced no stress using this system, because they did not perform any tasks during tasting and concentrated on providing feedback through the intensity meter.

To elucidate the effect of aging on taste perception, we compared the perceived salty taste intensity on the whole tongue over time in 31 older and 43 young adults. We hypothesized that older adults’ perceptions of a salty taste intensity would slowly increase and their perceptions of a salty taste intensity would generally be lower than those of young adults.

## Results

### Cup tasting test (experiment 1)

**Figure 1:**
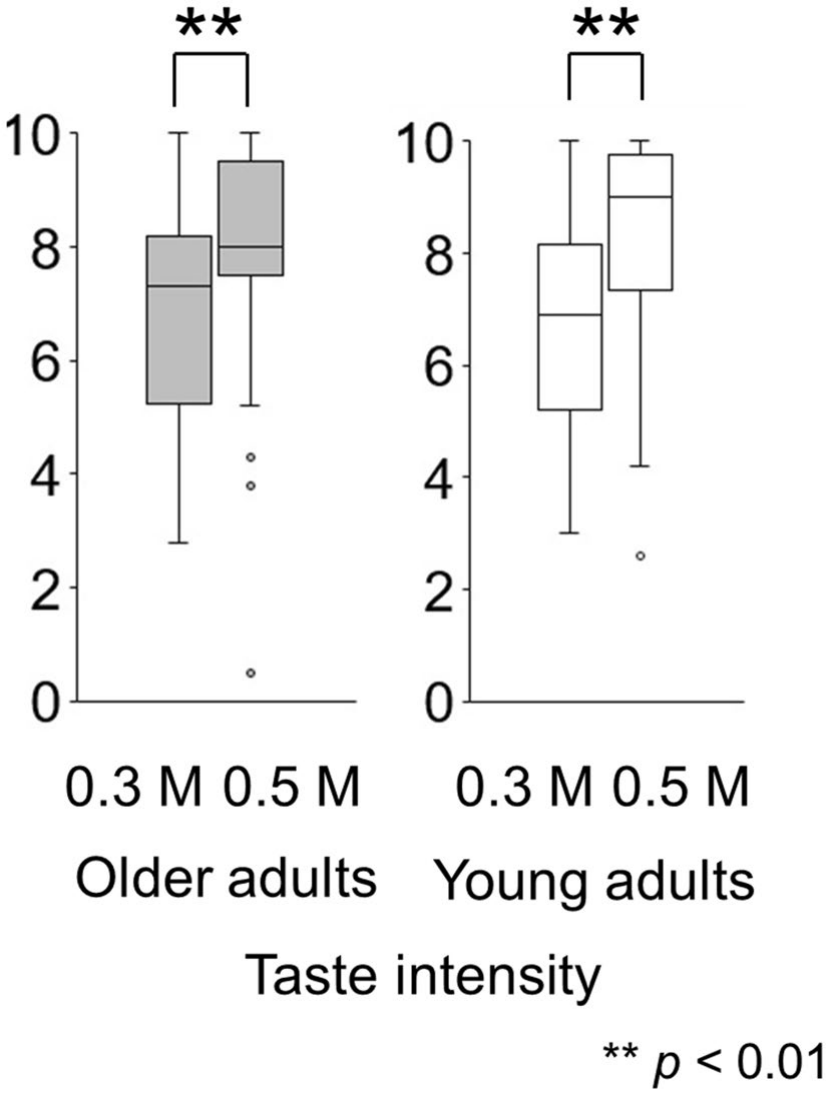
A difference in salty taste intensity between 0.3 M and 0.5 M solutions in a cup tasting test (experiment 1). Participants perceived the taste intensity of 0.5 M NaCl as significantly higher than that of 0.3 M for both older adults and young adults (*p* < 0.01).

**Figure 2:**
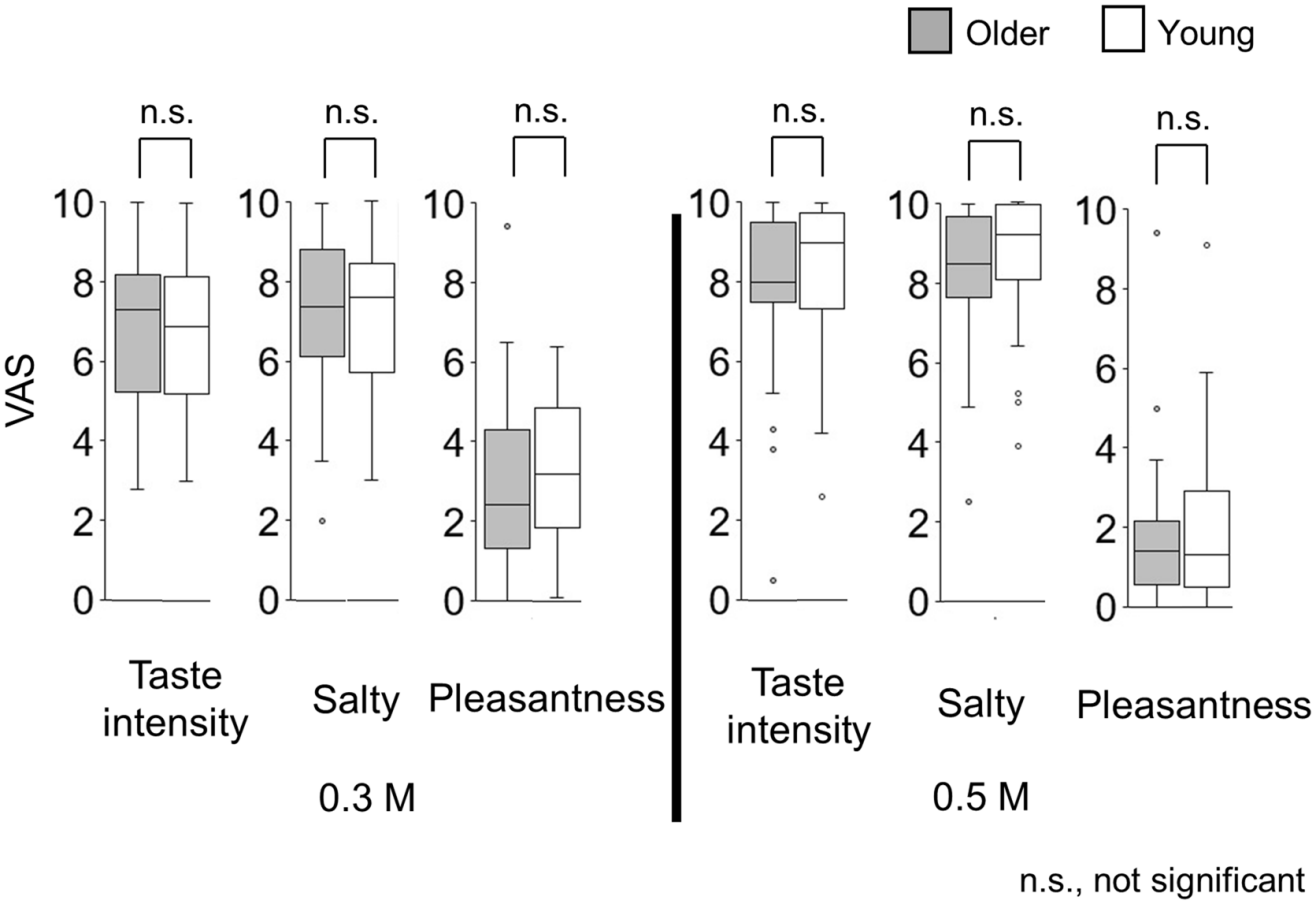
Intensity ratings of the cup tasting test (experiment 1). Participants sipped the solution from cups, hold it on the tongue with no tongue movement, gargling, or swallowing, and spit it out after 4 s, washed taste by distilled water, and rated static salty taste intensity and pleasantness on a provided paper sheet. No significant differences were observed between older and young adults. Older adults, n = 31; young adults, n = 43.

The results of the taste evaluations using the cup are shown in Figs. 1 and 2.

Paired tests showed that participants perceived the taste intensity of 0.5 M NaCl as significantly higher than that of the 0.3 M NaCl solution (older adults *p* = 0.003, young adults *p* < 0.001) (Fig. 1). Participants perceived the stimulus and differentiated between the two concentrations.

No statistically significant differences between older adults and young adults were detected for taste intensity, salty taste intensity, or pleasantness (Fig. 2).

### Time-intensity sensory evaluation (experiment 2)

**Figure 3:**
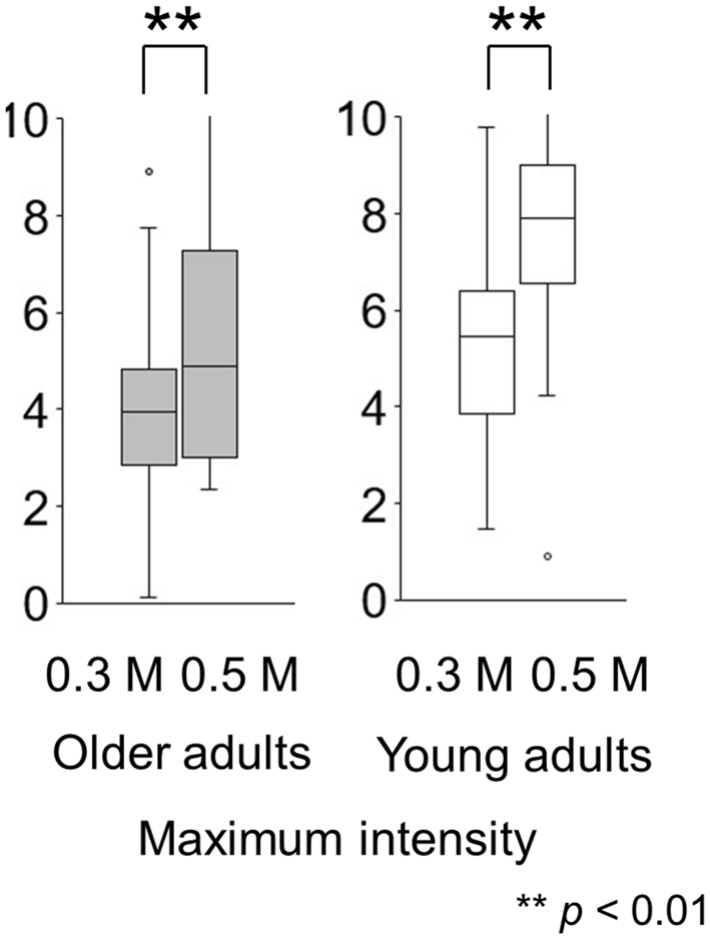
A difference in maximum taste intensity between 0.3 and 0.5 M solutions in time-intensity sensory evaluation (experiment 2). Participants perceived the taste intensity of 0.5 M NaCl as significantly higher than that of 0.3 M for both older adults and young adults (*p* < 0.01).

Paired tests showed that participants perceived the taste intensity of 0.5 M NaCl as significantly higher than that of the 0.3 M NaCl solution (older adults *p* = 0.002, young adults *p* < 0.001) (Fig. 3). Participants perceived the stimulus and significantly differentiated between the two concentrations.

**Figure 4:**
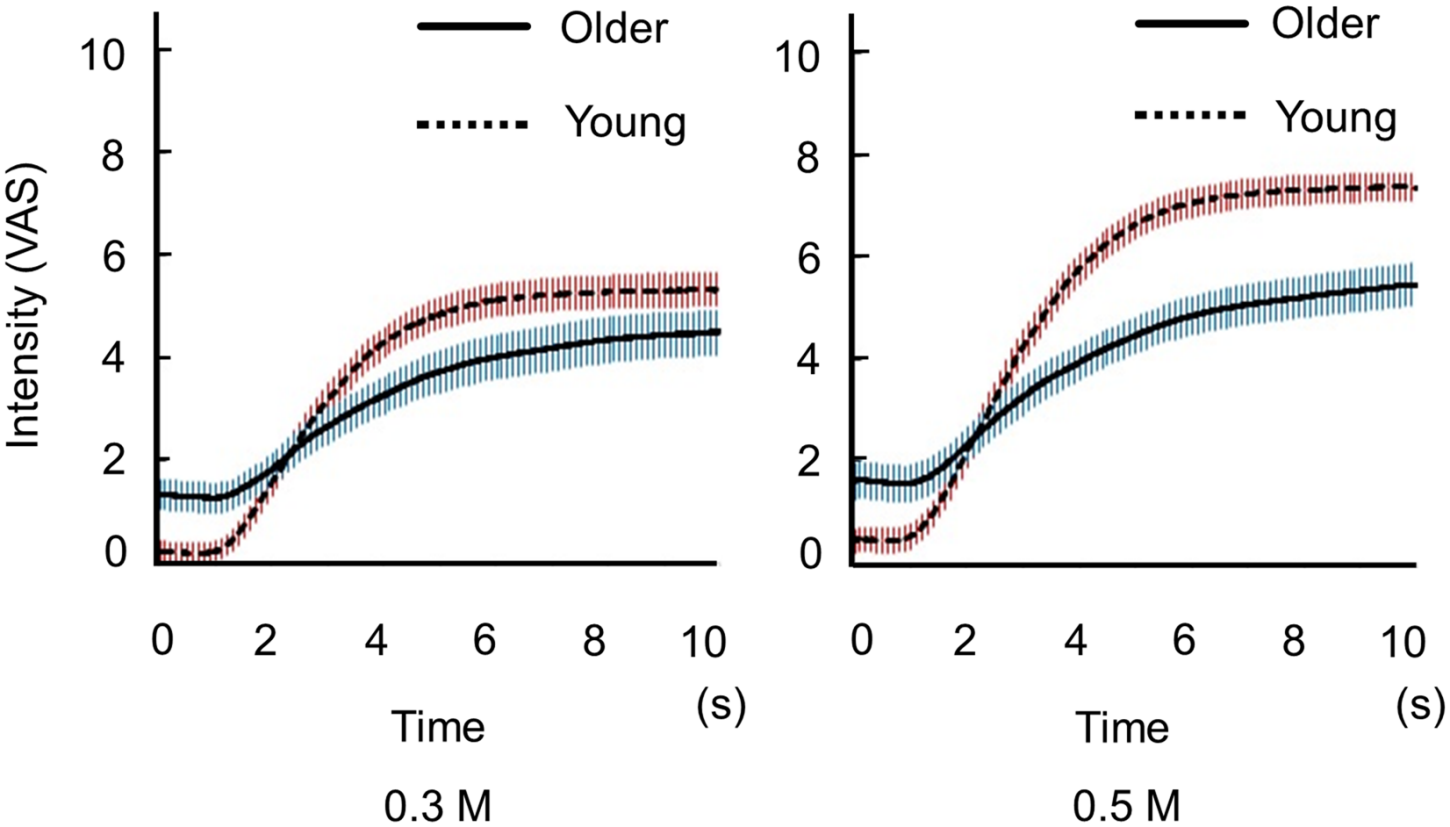
Time-intensity sensory evaluation of the salty taste intensity for all participants (experiment 2). The solutions were delivered to participants’ tongue through a custom-made delivery system while they recorded dynamic taste intensities on a hand-held time-intensity sensory evaluation meter. First, the 10 pairs of 0.3 M NaCl for 10 s and distilled water for 10 s were delivered in a blocked design. Next, 0.5 M solution was administered using the same method. We checked all time-intensity profiles and excluded profiles that included intensity meter operational errors. Then, the average of the replicated measurements of all profiles were calculated for each condition (figure shows mean ± 1 SEM). Older adults demonstrated slower rates of change in perceiving intensity of a salty taste, and lower perceived taste intensities than young adults. Older adults, n = 31; young adults, n = 43.

**Figure 5:**
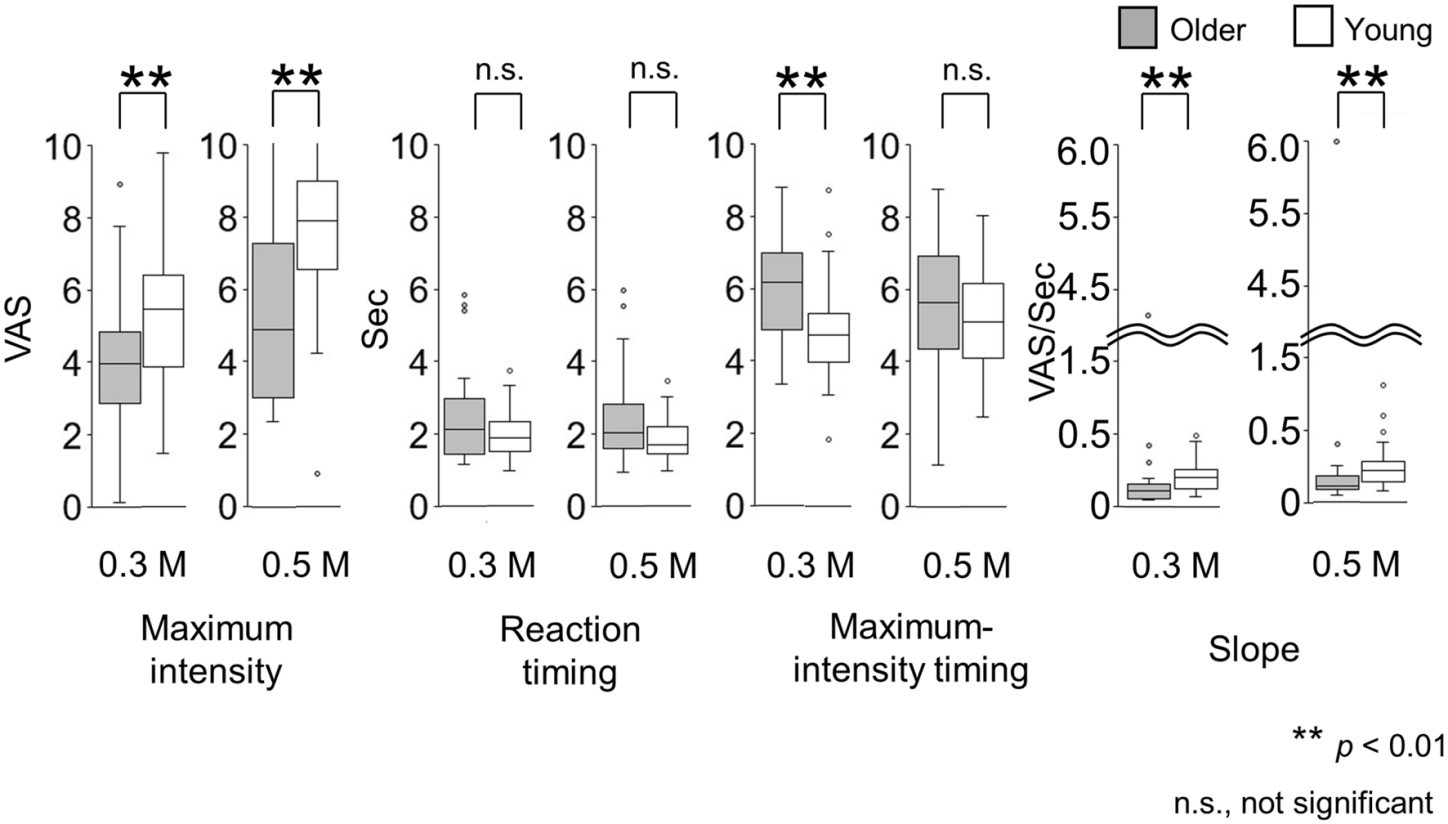
The features of the time-intensity profiles: Maximum intensity, reaction timing, maximum-intensity timing, and slope for the time-intensity sensory evaluation used for further statistical analysis (see Fig. 11) (experiment 2). Older adults rated significantly different maximum-taste intensities for both concentrations, compared with young adults. No significant differences in reaction timings were observed between older and young adults. The maximum-intensity timings were significantly different between older adults and young adults for 0.3 M. The slope for older adults was significantly different from that for young adults for both concentrations. Older adults, n = 31; young adults, n = 43.

The time-intensity profiles of the sensory evaluations and the data for the defined features are shown in Figs. 4 and 5, respectively. Older adults’ perceptions of salty taste intensity changed at a slower rate (slowly increased) and yet remained lower than those of young adults.

#### Maximum intensity

For 0.3 M NaCl, the median of the maximum intensities in older adults was 3.94 (Q1 to Q3, 2.85 to 4.81) and it was significantly different from that of young adults (5.45 [3.85 to 6.39]) (*p* = 0.004). The same result was found for 0.5 M (4.89 [3.00 to 7.29] and 7.91 [6.57 to 9.00]) (*p* < 0.001).

#### Reaction timing

The reaction timing of older adults was not significantly different from that of young adults.

#### Maximum-intensity timing

There were significant differences between older and young adults for 0.3 M NaCl (*p* = 0.001).

#### Slope

The slopes for both concentrations were significantly lower for older adults than for young adults. For 0.3 M NaCl, the slope for older adults was 0.11 (0.06 to 0.15) visual analogue scale (VAS) per second (s), which was significantly different than the 0.20 (0.12 to 0.26) VAS/s for young adults (*p* < 0.001). For 0.5 M NaCl, the slope for older adults showed a slower increase in taste intensity (0.11 [0.09 to 0.18] VAS/s) than that for young adults (0.22 [0.14 to 0.28] VAS/s) (*p* < 0.001).

#### Questionnaire ratings following time-intensity sensory evaluation

**Figure 6:**
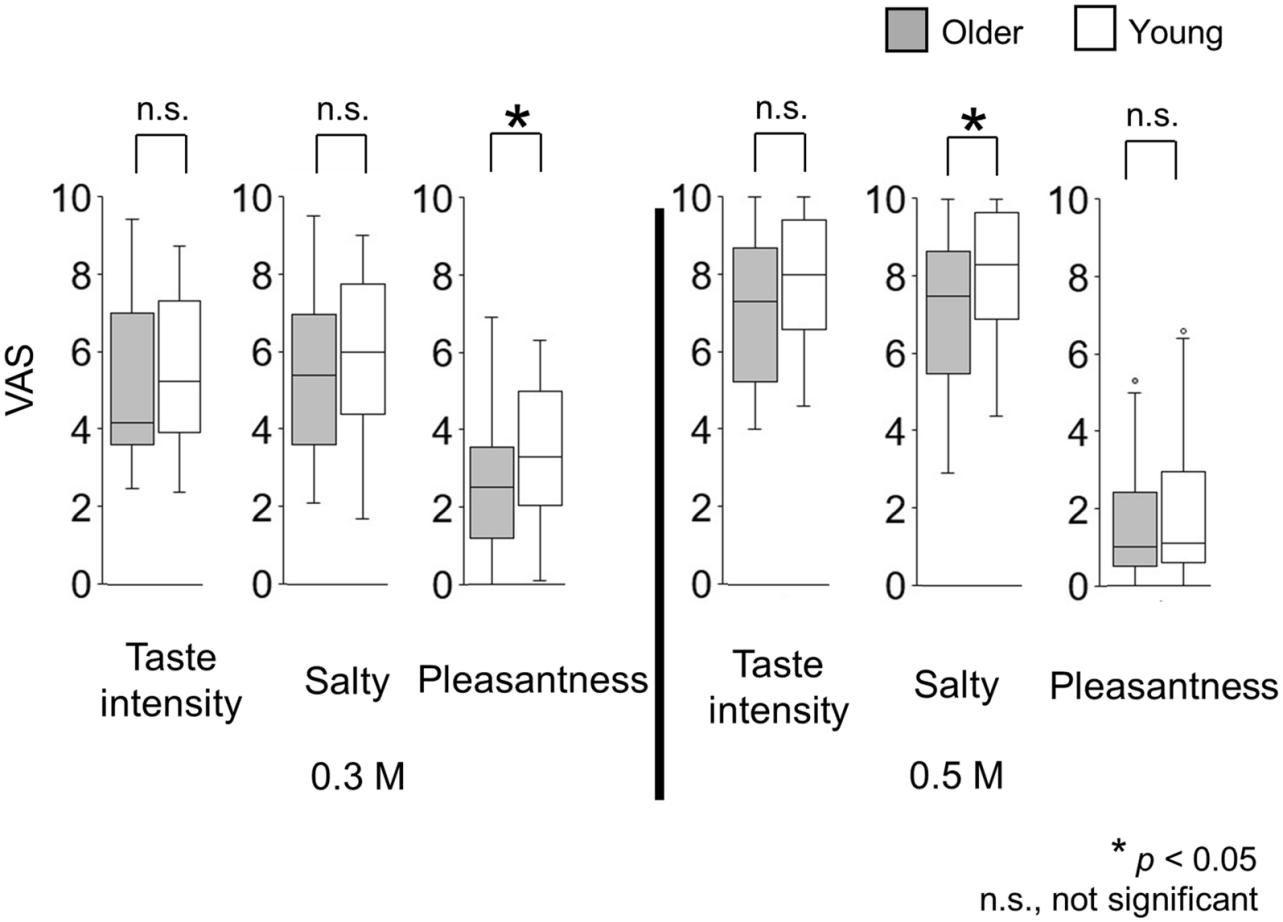
Questionnaire ratings following time-intensity sensory evaluation (experiment 2). The visual analogue scale scores on the questionnaire recorded immediately after the time-intensity sensory evaluations. Participants reported the taste intensity, salty taste intensity, and pleasantness perceived during the time-intensity sensory evaluation under standardized taste delivery conditions. These results show significant differences between older and young adults in perceptions of pleasantness for 0.3 M NaCl and for salty taste intensity for 0.5 M NaCl. Older adults, n = 31; young adults, n = 43.

The taste intensity, salty taste intensity, and pleasantness during the time-intensity sensory evaluations under standardized taste delivery conditions are shown in Fig. 6. There were significant differences between older adults and young adults in pleasantness for 0.3 M NaCl (*p* = 0.038) and in salty taste intensity for 0.5 M NaCl (*p* = 0.041).

## Discussion

This study is the first to characterize the time-intensity profiles of salty taste intensity in older adults. The results showed that older adults demonstrated slower changing rates in perceptions of intensities of salty taste and their perceptions of taste intensities remained lower than those of young adults (Fig. 4). The profiles showed that older adults rated the maximum-taste intensities and slopes significantly and differently from those of young adults for both 0.3 M and 0.5 M NaCl solutions (Fig. 5). In contrast, for the cup tasting condition, there were no statistically significant differences in VAS scores for taste intensity, salty taste intensity, and pleasantness between young adults and older adults (Fig. 2). Previous findings on differences in taste-intensity ratings of suprathreshold levels across age groups are inconsistent^19–25^. Participants in those studies were given solutions either by cups or syringes and the evaluation methods varied.

Therefore, we performed a time-intensity sensory evaluation using a system that delivered taste solutions in a standardized manner and recorded the perceived intensity in real time using a computer. There are no previous published studies on time-intensity sensory evaluations of taste using a standardized delivery of taste solutions on the tongue for older adults. In the two previous studies on time-intensity evaluations of salty solutions, participants recorded taste intensities while holding the salty solutions in their mouths; the studies involved 15 panelists (age unknown) and 12 young students^27,28^. Their time-intensity curves showed maximum intensities of approximately 4.0 and 5.2, respectively, and their maximum-intensity timings were between 6 and 8 s. Although our results are comparable with theirs, direct comparisons cannot be made because the concentrations and methods used were different. As for the maximum-intensity timing in our study, there were significant differences between older and young adults for 0.3 M NaCl (*p* = 0.001) but not for 0.5 M. This may be because the variation within the group was larger at 0.5 M than at 0.3 M (Fig. 5). Maximum-intensity timing (horizontal axis, Fig. 4) could vary because time-intensity profiles started to plateau. Not only the statistical results at the selected points, but also careful observation of whole profiles helped us to understand features of perception.

We realized that standardized conditions were important for evaluating subtle differences. Therefore, we used a system that could record data under standardized conditions to evaluate the differences between the groups^26^. The following measures were implemented: (1) dental techniques were used to maximize the stability and comfort of the intra-oral device that fitted on each participant’s dental arch so that participants could concentrate on tasting; (2) an intra-oral device delivered the taste solution as wide as possible to the whole tongue, including the anterior, lateral, and posterior tongue; (3) there was always a constant amount of solution on the tongue, and differences in saliva flow rate between participants did not affect the data; (4) participants did not swallow the solutions. Taste buds in the pharynx, soft palate, and gut were not stimulated so that repeated measurements were possible (i.e., 10 times for each solution for each participant in this study); (5) a time-intensity sensory evaluation meter linked to a synchronized taste solution delivery system allowed time-intensity profiles to be recorded in high temporal resolution and in real time. As a result, we obtained consistent data that allowed us to detect tongue sensations and differences between older adults and young adults.

We also realized that repeated measurements would be a more valid way for evaluating salty taste intensity for each participant. A previous study indicated that time–intensity curves of bitterness measured in the first session, perceived intensity was significantly lower in the first than in the second, third, and fourth trials, and that time courses of perceived intensity did not differ among the second, third, and fourth trials^29^. They proposed that in order to obtain reliable performance in time–intensity evaluation, untrained participants should be provided a training trial using warm-up sample before staring the test trials^29^. In our study, warm-up samples were given at the beginning of experiment 2 (see “Materials and methods” section), and strange profiles varied across 10 repetitions in each solution for each participant. Therefore, by implementing repeated measurements, we could observe, remove strange profile, and calculate the features for each participant without including strange values by chance.

Furthermore, using a standardized system allowed us to evaluate and directly compare real-time feedback on taste intensities according to age. In this system, reaction time measurement, together with time-intensity taste sensory evaluation, was possible. Older adults did not take longer to recognize a salty taste, but their perception of taste intensity increased more slowly, and remained lower than that of young adults (Figs. 4, 5). To our knowledge, there are no reports in the literature of such data for older adults.

The comparison of absolute time values is difficult because the time sequence can be affected by the timing and solution delivery methods. For example, absolute values of reaction times in previous studies were approximately 2.3 s^28^ and 0.6–1 s^26,30–33^; the maximum-intensity times were approximately 3.8 s^31^ and 6.12–8.12 s^27,28^. Previous studies indicate that the simple reaction times for hearing and optic testing of 65-year-olds are approximately 10% and 16% longer, respectively, than those of 25-year-olds^34,35^. One study that used taste disks demonstrated that older adults showed age-associated deterioration in taste-detection thresholds, whereas the somatic sensations of the tongue were well retained^1^. These studies show that the effects of aging are slow and varied. Given these previous findings, our direct comparison of time-intensity sensory profiles of older adults and young adults under the same conditions is useful in expanding the understanding of the dynamic features of taste perception in older adults.

One limitation of this study was that the number of older adults who participated was smaller than that of the young adults. Despite employing various recruitment methods, we found it quite difficult to recruit older citizens, particularly those > 75 years old. Additional studies on taste with larger samples of late- and older-age participants are needed. It is interesting to note, however, that 60–69-year-olds’ salt intake has been greater than that among 70 + -year-olds in Japan over the past 8 years^36^. Therefore, the data in the present study are useful for efforts to understand why this situation exists in Japan: people who ingest less salt may be living longer or people over 70 years old may be receiving nutrition guidance from family physicians or social welfare.

Another potential limitation of this study was the differing sex ratios between the younger and older groups: 50% female in the former versus 40% female in the latter. However, additional recruitment of older adults at this point was not allowable due to coronavirus disease (COVID-19) pandemic. Therefore, to determine the possible impact of this imbalance on our results, we randomly excluded six female participants from the younger group so that both groups were 40% female. We then made 10 sets of such data and analyzed them. The results showed that all time-intensity profiles evidenced similar patterns. Statistical differences in maximum-intensity timing for 0.3 M solutions were nonsignificant, while other parameters showed the same levels of significance as the current data. It should be noted that maximum-intensity timing could vary as time-intensity profiles start to plateau (see Fig. 4).

A third limitation of this study is the lack of clarity about the relative importance of medication and age for the observed group differences. The review article by Schiffman reported that functional measurements of chemosensory processes have not yet been performed in systematic well-controlled clinical trials that evaluate the side effects of a wide range of medications so the incidence of drug-induced chemosensory disorders is unknown. However, based on information at that time, the incidence of adverse chemosensory effects from drugs depends upon the specific medication with an average of 5% across most medications but up to 66% for the drug eszopiclone used to treat insomnia. Further, exposure of the tongue to the diuretic amiloride, which blocks the epithelium sodium channel, reduced the taste intensity of NaCl^37^. In our study, eight out of 31 older adults had taken medication for high blood pressure, hyperlipidemia, reflux esophagitis, and diabetes. Seven medicines for six older adults in our study are listed as drugs that elicit taste complaints (Table 1 in the reference^37^). All participants in our study were able to distinguish between the two NaCl solutions used, and data distribution of them did not show any special feature according to with or without medicine. Our data showed nonsignificant differences in the subtle variations observed in maximum intensity between older adults who were taking antihypertensive drugs (four people) and those who took none (23 people); neither of maximum intensity nor slope between older adults who were taking medication (eight people) and those who took none (23 people) (*p* > 0.05, The Mann–Whitney U test). This might be because eszopiclone or amiloride were not taken by our participants. The adverse reaction by medication was not significant in our study, we should always consider small effects of medication on taste are possible.

The fourth potential limitation was that the investigators were not blind to the solution used. To avoid investigator bias, therefore, all data were checked and analyzed repeatedly by a statistician, the P.I., an engineering specialist, and multiple data collectors. We respected and employed almost all the data, and only excluded the data affected due to difficulties using the intensity meter and the outliers identified by statistical methods proposed by Tukey^38^ (please refer to the “Materials and methods” section for details).

A fifth potential limitation was that we did not train the participants or give them any information about the solution. Thus, intensity estimates may have been higher in the cup-sipping stimulation (experiment 1) compared with those recorded during and immediately after the time-intensity sensory evaluation (experiment 2). Information gleaned from a pilot study, from participants’ comments, and from researchers’ observations in this study indicated that the reason for the higher intensity estimates in the cup-sipping stimulation was mainly because of the participants’ first impression of the salty solution. They performed the cup tasting test first, and then the time-intensity sensory evaluation. Thus, participants tasted the 0.3 M solution for the first time in the cup tasting test; many felt it was ‘salty’ and gave it a high rating. In the time-intensity sensory evaluation, participants focused diligently on perceiving the taste intensity as it was. Other factors such as longer stimulation time, repeated stimulations (10 times) of a salty taste, and sitting position vs supine position did not substantially impact participants’ evaluations.

Finally, the standardization of the artificial stimulation method may lack ecological validity, given that consumers drink from cups in real-world situations. However, the cup tasting experiment (experiment 1) was able to provide ecological validity as well as offer a way to measure both taste intensity and taste pleasantness. Furthermore, the approach used in this study can inform future experimental studies involving older adults with a control group among ordinary citizens. First, participants did not need to swallow the solutions; therefore, the risk of aspiration was quite low. In fact, no participant experienced aspiration in this study. Second, participants did not need special training. In a previous study on time-intensity scaling with taste judgments, young participants were trained to identify taste solution concentrations for 9.0–17.25 h, then performed the experiment for 0.75–1.25 h for one taste. After participants had learned to estimate the intensity directly in mM concentrations, they held taste solutions in the mouth and recorded their perceived taste intensities by moving a fiber-tipped pen over a chart recorder^39^. Another study performed training 3 days a week for 2 months, and 15 panelists out of 25 volunteers were selected^27^. We appreciate that training participants serves to reduce inter-individual variability in sensory evaluations so that obtained data are more reliable and reproducible, and required panel size is not too large. In our study, however, we did not train the participants to maintain the motivation required to attend to the series of experiments, and to avoid participant bias by fatigue, especially for older adults. Therefore, it should be noted that our results reflect the special conditions of the study and may show large inter-individual variability. Considering these conditions, the careful investigations at both group and individual levels were very important in our study.

## Conclusions

These results are the first to demonstrate differences in the outcomes of salty taste intensity sensory evaluations in healthy older and young adults. The time-intensity profiles of older adults showed significantly different perceptions from those of young adults, quantitatively. Our findings indicate that older adults do not take longer than young adults to recognize a salty taste, but after the initial awareness, their perception of taste intensity increases slowly and remains lower than that of young adults. This suggests that older adults should be aware of the tendency to add more salt to their food to compensate for their low perceptions of salty taste. We suggest, moreover, that older adults savor and chew sufficiently during eating to optimize perceived salty taste. In the future, these findings can provide a reference for taste intensity experienced by ordinary citizens; our system and results can be usefully integrated into clinical follow-up examinations. For example, assessing taste perception that decreases and recovers during and after COVID-19 infection or tongue cancer will support patients’ quality of life.

## Materials and methods

### Participants

Eighty-one healthy adults participated in this study. The older adults comprised 36 individuals (22 male, 14 female; age, 60–81 years [65.3 ± 5.4 standard deviation, SD]; body mass index [BMI], 22.4 ± 2.6 [SD] kg/m^2^). The young group consisted of 45 adults (22 male, 23 female; age, 21–39 years [25.5 ± 4.2 SD]; BMI, 21.5 ± 2.2 [SD] kg/m^2^). Because this study was preliminary, we set the number of participants per group tentatively. For reference, we calculated the average number of participants in previous studies: 33.7 (18–88 participants) in filter disk tests and cup tasting tests^1,3,9–11,20,21,24,25^, and 11.2 (9–15 participants) in time-intensity sensory evaluations^27,28,39^. Recognizing that the same participants will participate in our next experiment—the fMRI research—we set our target sample size to 40 participants for each group, following a recent study of sample sizes for task-fMRI in taste research^40^. Participants were recruited through advertisements around Tokyo Dental College and via a large-scale online registry system, the Integrated Registry of Orange Plan (IROOP®).

We excluded individuals from this study if they met at least one of the following criteria: (1) tobacco smoker; (2) having a known taste, smell, psychiatric, or neurological disorder; (3) inability to distinguish between the two NaCl solutions used in this study; (4) older adults suspected of having dementia with a Mini-Mental State Examination score ≤ 22^41^; (5) difficulties using the intensity meter during time-intensity recording. Five older adults and two young adults were excluded for the latter reason; therefore, data from 74 participants were analyzed. The older adults in this final sample comprised 31 individuals (18 male, 13 female; age, 60–81 years [65.0 ± 5.5 SD]; BMI, 22.3 ± 2.5 [SD] kg/m^2^); and the young group consisted of 43 adults (21 male, 22 female; age, 21–39 years [25.0 ± 3.6 SD]; BMI, 21.5 ± 2.3 [SD] kg/m^2^). Eight older adults had taken medication for high blood pressure, hyperlipidemia, reflux esophagitis, and diabetes. The package inserts of these medications stated that the incidence of adverse reactions associated with taste was less than 1%. No participants with medication was excluded because they distinguished between the two NaCl solutions used in this study. The participants were ordinary citizens with no special training or experience. The study was conducted according to the Declaration of Helsinki on Biomedical Studies Involving Human Subjects; and Ethical Guidelines for Medical and Health Research Involving Human Subjects by Ministry of Health, Labour and Welfare; and Ministry of Education, Culture, Sports, Science and Technology, Japan. The institutional review board of Tokyo Dental College approved the study (No. 676), and written informed consent was obtained from the participants.

### Taste solutions

The stimuli consisted of 0.3 and 0.5 M NaCl solutions prepared with distilled water. No other taste qualities were implemented. According to the results of two pilot studies conducted by the authors (five young participants each), the 0.1 M and 0.2 M solutions were removed and 0.3 and 0.5 M were chosen in consideration of the balance of perceived intensities, the ability to differentiate between the two concentrations, and the possibility of rinsing them out of the tongue. The concentrations of 0.3 and 0.5 M were based on estimations for fish cakes and seawater/Japanese pickles, respectively. The Wilcoxon signed-rank test for nonparametric and paired data was performed to measure the reported differences in salty taste between the 0.3 M and 0.5 M solutions. We used this test to evaluate both taste intensity in the cup tasting test and maximum intensity in the time-sensory evaluation.

Distilled water was used as the control and for rinsing salty solutions. All solutions were kept at 25 °C.

### Experimental design

To systematically investigate perceptions of taste in the mouth and brain, and thereby obtain consistently high-quality data, we have used the same participants throughout our three-step study. The three sequential experiments/steps were:

- Experiment (1) cup tasting test (static).
- Experiment (2) time-intensity sensory evaluation (dynamic).
- Experiment (3) functional magnetic resonance imaging (fMRI).

In this paper, we reported on experiments (1) and (2), taste perception in the mouth, or so-called sensory evaluation. We performed both experiments in the same day. The participants tasted the salty solutions in two ways: (1) sipping from a cup, and (2) having a salt solution delivered directly into the mouth through custom-made delivery system (the same system will be used in the fMRI experiment). The details of each procedure are described in the following sections.

#### Cup tasting test (experiment 1)

Because the cup tasting test was simpler and more natural than the time-intensity sensory evaluation, it was the more appropriate method to use for participants’ first experience of assessing and recording taste intensity, especially for older adult citizens (non-panelists). Each participant performed the cup tasting test once for each of the solutions (0.3 M and 0.5 M).

The method entailed sipping the salt solution from the cup, holding it in the mouth, spitting out the solution after 4 s, washed taste by distilled water, and rating the taste intensity and pleasantness on a provided paper sheet. Each participant tasted 2 mL of each solution in a paper cup with no label. The participant was instructed to sip all of each solution, hold it on the tongue with no tongue movement, gargling, or swallowing, and spit it out after 4 s. Tasting for 4 s is the same stimulation duration that we will use in our future fMRI study. The rationale behind the 4 s for a fMRI with brain network analysis is ‘sufficient stimulation in as short a time as possible’. In a previous study, the maximum-intensity timing for 0.1 M NaCl (lower concentration than 0.3 and 0.5 M in the current study) was 3.8 s^31^; therefore, we considered 4 s an ideal duration for our study.

During those 4 s, participants kept the solution on their tongue; neither the floor of the mouth nor the soft palate was stimulated. This procedure was used to maintain consistency with the methods used in experiments (2) and (3), in which the solution is only in contact with the tongue. While this presented challenges during the cup tasting test, participants did their best and no problems were reported during this experiment. Taste perceptions emanating from the oral floor and the soft palate were not reported.

**Figure 7:**
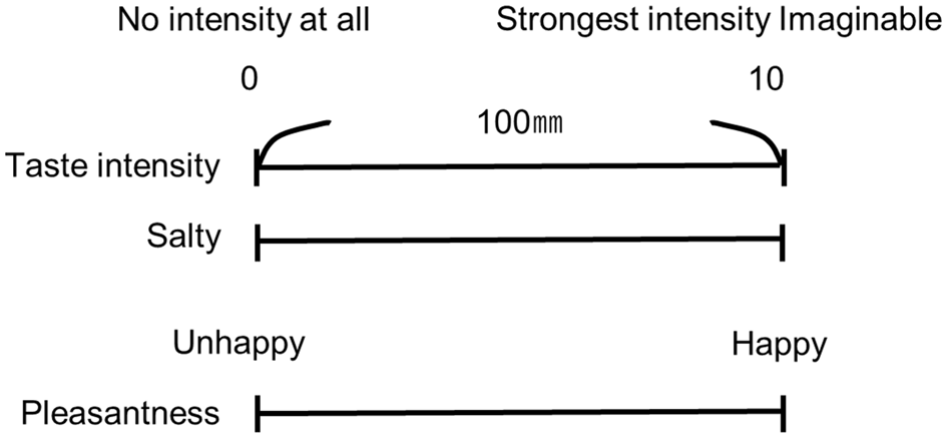
Visual analogue scale (VAS). A 100-mm straight horizontal line was used to represent the VAS. The poles of the scale were ‘no intensity at all’ and ‘strongest intensity imaginable’.

Participants rated the intensity of the overall taste (sum of all taste qualities perceived), salty taste intensity, and the pleasantness of the solution on a 0–10 VAS; ‘0’ represented ‘no intensity at all’ and ‘10’ represented the ‘strongest intensity imaginable’ (Fig. 7). Participants were provided with distilled water in another paper cup to completely rinse the residual taste before receiving another solution.

Only NaCl was used, and all participants were blinded with regard to taste quality. Given the known sweet taste of a 0.001 M NaCl solution^19,39^, it was possible that participants might perceive other taste qualities, too. Therefore, we asked them to rate both the overall taste (sum of all taste qualities perceived) and the salty taste alone.

At the end of cup tasting test, participants were provided with distilled water in another paper cup to completely rinse the residual taste before time-intensity sensory evaluation (experiment 2).

#### Time-intensity sensory evaluation (experiment 2)

**Figure 8:**
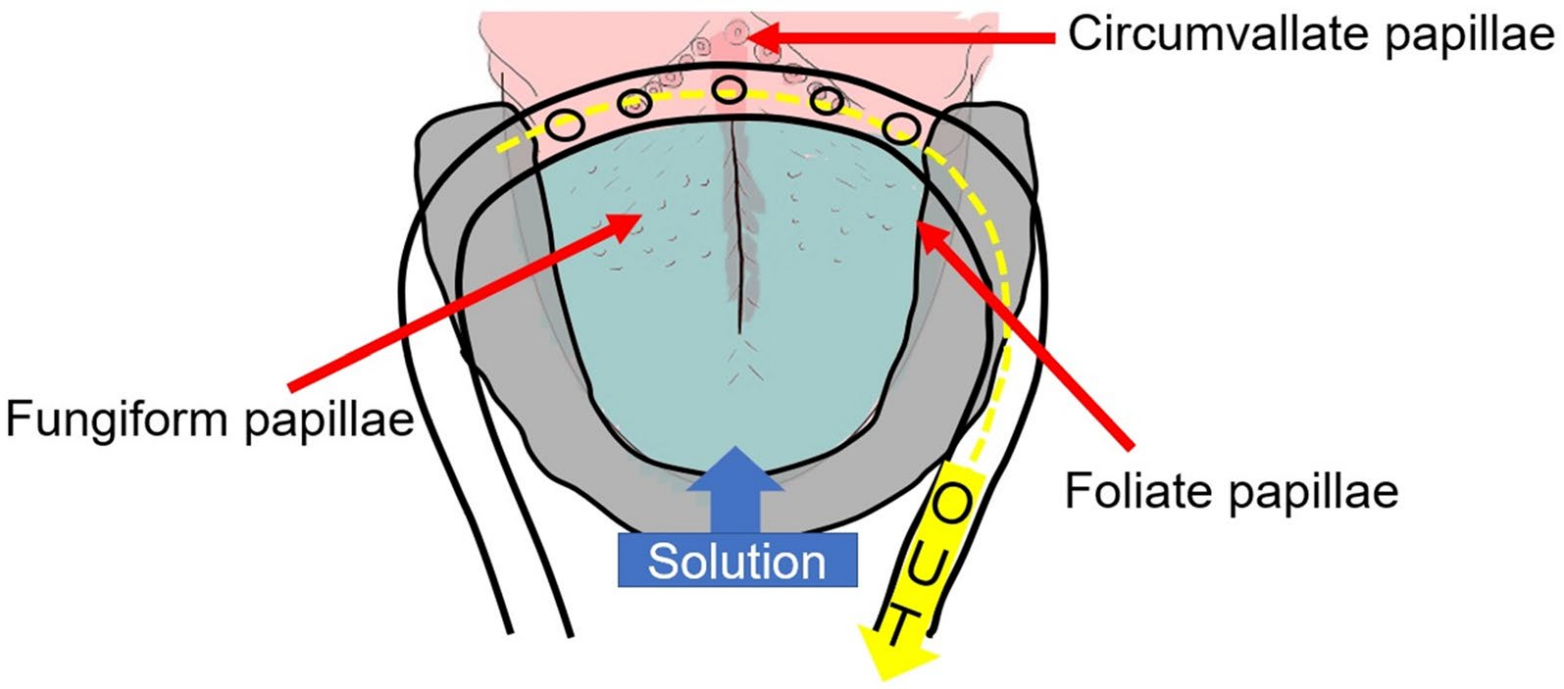
The intra-oral device for time-intensity sensory evaluation (experiment 2). This device consisted of upper and lower dental splints with inlet tubes attached at the front. The solution flowed to the dorsal and lateral sides of the tongue covering the fungiform and anterior half of the foliate papillae. A suction tube placed at the back of the mouth removed the solution. Please also see Figures 1–3 in Goto, et al.^26^.

During the time-intensity sensory evaluation, the solutions were delivered to each participant using the taste solution delivery system under standardized conditions^26^. The intra-oral device was tailored to each individual’s dental arch and gagging reflex level. This ensured that the solution flowed onto the dorsal and lateral sides of the tongue, covering the fungiform and anterior half of the foliate papillae. An adjustable suction tube placed at the back of the mouth removed saliva and the solution, so participants did not have to swallow the solution (Fig. 8). The extra-oral solution control system was operated by an original computer program and provided a continuous steady flow of solution to the whole tongue. The solution flow rate was set at 110 mL/min and monitored by flow meters so that the participant experienced no tactile sensations on his/her tongue^26^. At the beginning of experiment 2 for each participant, first, we spent about five minutes to check the system by delivering distilled water. During that, tongue of the participant was washed by distilled water. Second, warm-up samples, i.e., taste solution and water were delivered to participant’s tongue once for each solution at the beginning of time–intensity evaluation (experiment 2), then, formal test below started. The participants knew the same solution experienced at the experiment 1 will be delivered while did not know neither the component nor intensity of those taste solutions.

**Figure 9:**
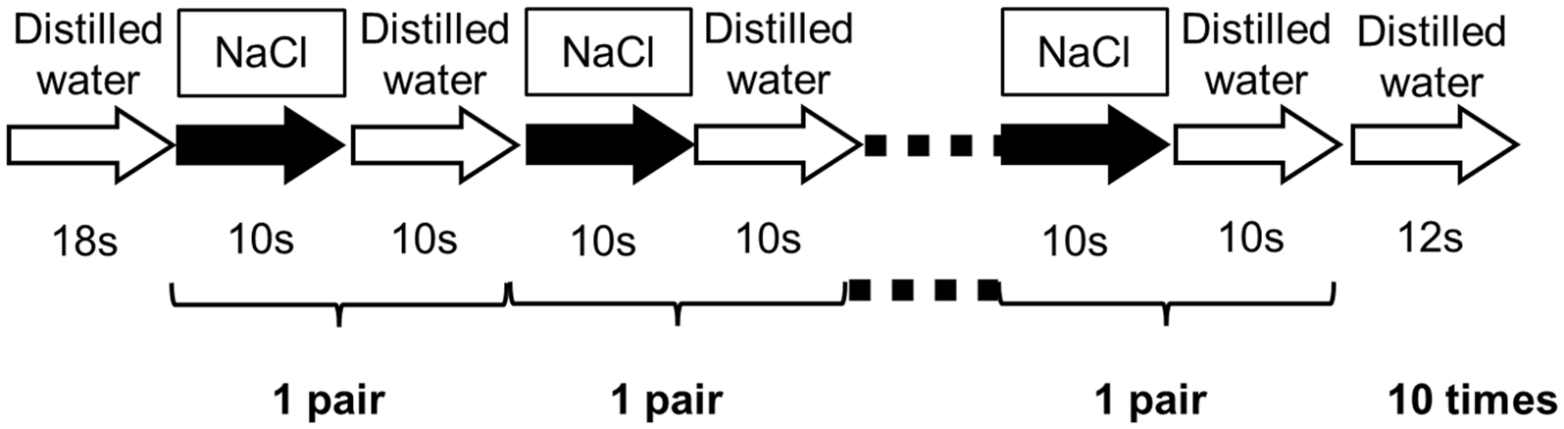
Experimental design (experiment 2). The experiment was carried out for 0.3 M and 0.5 M NaCl solutions. Each session started with distilled water, followed by 10 pairs of NaCl for 10 s, distilled water for 10 s, and, finally, distilled water.

We used a block design. The experiment started with 0.3 M NaCl (a lower concentration solution). The session began with distilled water for 18 s. This was followed by 10 pairs of NaCl for 10 s, distilled water for 10 s, and finally, distilled water for 12 s. We administered the 0.5 M solution using the same method (Fig. 9). Only one concentration within one block was used. We avoided complicated methods for older adults so they could more easily concentrate on their evaluations of taste intensity without tiring. The duration of 10 s was chosen on the basis of a study showing that the time to reach maximum intensity was 3.8 s for a salty solution and 6.0 s for a sweet solution^31^. The 10-s stimulation time would thus be long enough to ensure the maximum-intensity timing for 0.3 M and 0.5 M for both young and older adults.

In this study, we also recorded the decline in perceptions of salty taste intensity at time-intensity sensory evaluation but do not report those data in this article because that decline was not physiological but rather artificial, owing to the distilled-water rinse (Fig. 9). We continuously delivered the taste solution for 10 s, during which we acquired a dynamic taste profile, to ensure maximum intensity was reached and then intensity declined. Furthermore, it was essential to confirm that the salty taste was completely washed out by the distilled water; washing away the stimulus is important for the future fMRI experiment.

**Figure 10:**
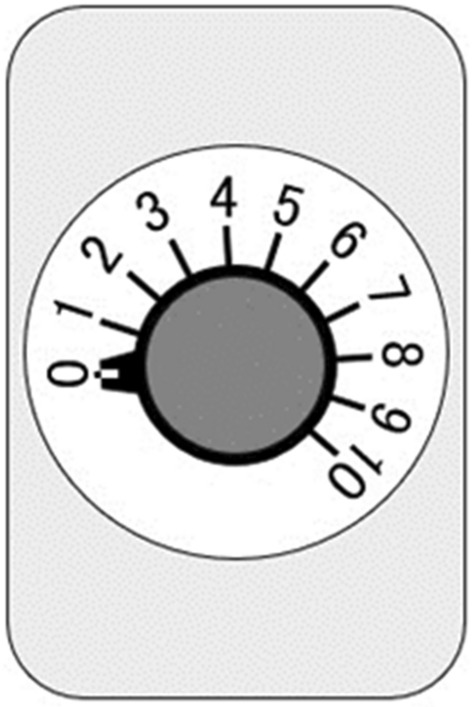
The rotary dial on a hand-held time-intensity sensory evaluation meter used during the administration of the taste solution (experiment 2). The scale on the dial corresponded to a visual analog scale, ranging from 0 (no taste) to 10 (strongest taste imaginable).

Participants recorded the overall perceived taste intensity using the rotary dial on a hand-held time-intensity sensory evaluation meter during the administration of the taste solution. The rotary dial used in the study was internally developed by Goto et al*.*^26^, as was the oral device and the solution delivery system. The rotary dial consisted of a knob (17 mm in diameter) and a variable resistor (ALPHA RD902F, 500 kΩ, 9 mm). The scale on the dial corresponded to a visual analog scale, ranging from 0 (no taste) to 10 (strongest taste imaginable) (Fig. 10). We instructed participants to start turning the dial as they perceived a taste and to position the dial according to the perceived taste intensity in their mouth. The analog signal generated by the rotary dial was transferred to a microcontroller (Arduino Uno R3) and was converted to a digital signal by a computer. A challenge in designing this analog device was its sensitivity. We constructed a device that continuously transmitted an electrical signal and plotted a graph of the intensity against time for each participant so that both the mechanical and human errors could be checked during the experiments. A computer program was written specifically for this study protocol. The time-intensity evaluation meter was synchronized with a computer-controlled taste solution delivery system connected to an intra-oral device. This design allowed us to monitor the participants’ perceptions in real time.

#### Questionnaire ratings following time-intensity sensory evaluation

Immediately after the time-intensity sensory evaluations, participants used a questionnaire to rate taste intensity, salty taste intensity, and pleasantness perceived during the time-intensity sensory evaluation under standardized taste delivery conditions. They reported VAS scores using another questionnaire (Fig. 7).

The experimental conditions were fixed. Participants started the ratings at approximately 5:30 pm. All participants were tested in the same room; the room temperature was maintained at 24.1 ± 1.2 [S.D.] °C.

### Data analyses and statistics

Statistical analyses were performed using R software 3.4.1 (www.r-project.org). Statistical significance was set at *p* < 0.05.

#### Questionnaires

Shapiro–Wilk tests were performed to test the normality of the data distribution. Following the results, nonparametric tests were applied. The Mann–Whitney U test was used to test for significant differences between the older and young adults in intensity ratings of overall taste, salty taste, and pleasantness. We had not performed multiple comparisons as we compared young group and older adults group according to our hypothesis.

#### Time-intensity sensory evaluation

The time-intensity profiles of the taste solutions administered for 10 s were analyzed using MATLAB R 2019a (The MathWorks, Inc., Natick, MA, USA). We checked all time-intensity profiles and excluded profiles that included intensity meter operational errors. As a result, 0.9% (0.3 M young adults), 2.1% (0.5 M young adults), 5.2% (0.3 M older adults), and 6.8% (0.5 M older adults) of the profiles were excluded. Then, the average of the replicate measurements of all profiles and standard error of the mean (SEM) were calculated for each condition.

**Figure 11:**
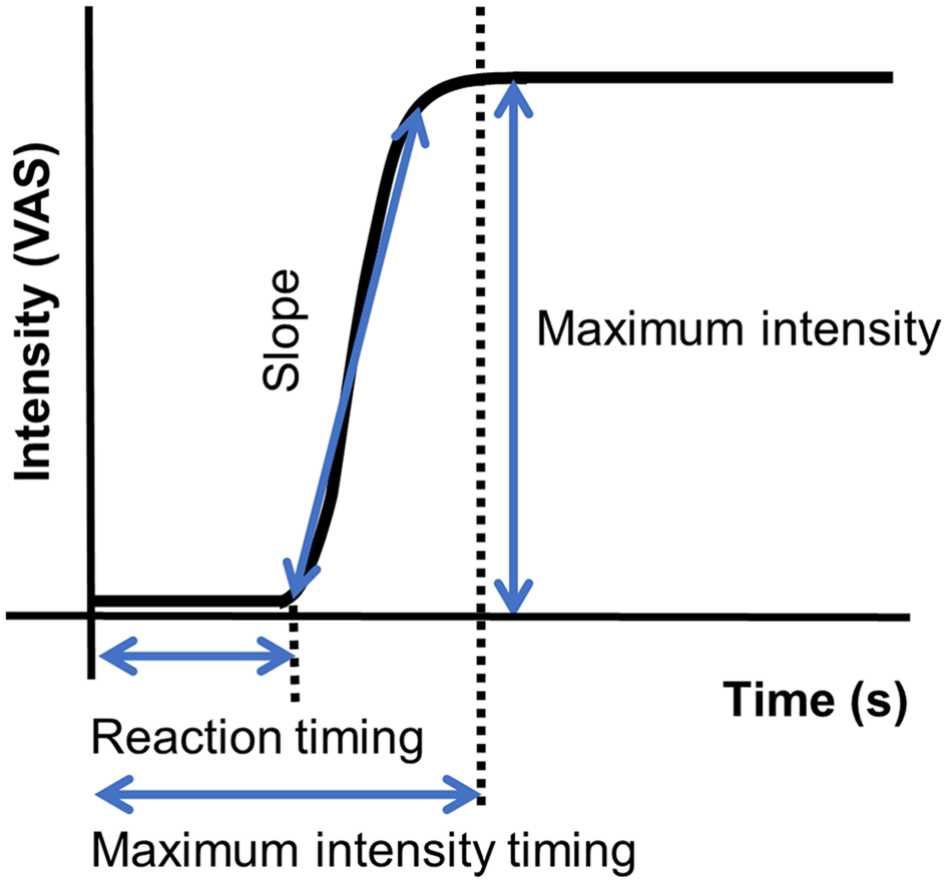
Features defined on the time-intensity profile (experiment 2). Maximum intensity is the mode of the smoothed intensity larger than 80% of the maximum smoothed intensity above the baseline; that is, the highest and longest plateau on the profile; reaction timing is the timing at which the value of taste intensity starts to become larger than baseline intensity; maximum-intensity timing is the timing in milliseconds for the intensity curve to plateau; the slope is the change in VAS score (on a 0–10 scale) per s.

The features of the time-intensity profiles used for further analyses included the following (Fig. 11): (1) maximum intensity (the mode of the smoothed intensity larger than 80% of the maximum smoothed intensity above the baseline; that is, the highest and longest plateau on the profile); (2) reaction timing (the time at which the taste-intensity value started to become larger than the baseline intensity); (3) maximum-intensity timing (the timing in milliseconds for the intensity curve to plateau); (4) slope (the best-fit straight line was calculated using linear regression from 10 selected time-intensity points)^26^.

The Shapiro–Wilk test was used to test the normality of the distribution. Following the results, nonparametric tests were applied. The outliers for each participant were identified as those outside the following interval: [Q1 − 1.5 · IQR, Q3 + 1.5 · IQR], where ‘Q’ stands for ‘quartile’ and ‘IQR’ stands for ‘interquartile range’^38^. Mann–Whitney U tests were used to investigate the differences between the groups. We had not performed multiple comparisons.

### Ethical approval

The methodology for this study was approved by the institutional review board of Tokyo Dental College (No. 676).

### Consent to participate

Participants provided written informed consent.

## Data Availability

The dataset generated and analyzed during the current study is available from the corresponding author on reasonable request.

## References

[1] Fukunaga A, Uematsu H, Sugimoto K (2005). Influences of aging on taste perception and oral somatic sensation. J. Gerontol. A Biol. Sci. Med. Sci..

[2] Methven L, Allen VJ, Withers CA, Gosney MA (2012). Ageing and taste. Proc. Nutr. Soc..

[3] Yoshinaka M (2016). Age and sex differences in the taste sensitivity of young adult, young-old and old-old Japanese. Geriatr. Gerontol. Int..

[4] Ministry of Health, Labour, and Welfare. National Health and Nutrition Survey Report 2018. https://www.nibiohn.go.jp/eiken/kenkounippon21/download_files/eiyouchousa/2018.pdf

[5] World Health Organization (WHO). T*he world health report 2002: Reducing risks, promoting healthy life.* World Health Organization (2002).

[6] World Health Organization (WHO). *Guideline: Sodium intake for adults and children.* World Health Organization (2012). 23658998

[7] World Health Organization (WHO). *Salt reduction.* World Health Organization. http://www.who.iny/news-room/fact-sheets/detail/salt-reduction (2020).

[8] Strazzullo P, D’Elia L, Kandala N-B, Cappuccio FP (2009). Salt intake, stroke, and cardiovascular disease: Meta-analysis of prospective studies. BMJ.

[9] Mojet J, Christ-Hazelhof E, Heidema J (2001). Taste perception with age: Generic or specific losses in threshold sensitivity to the five basic tastes?. Chem. Senses.

[10] Satoh Y, Seluk LW (1988). Taste threshold, anatomical form of fungiform papillae and aging in humans. J. Nihon Univ. Sch. Dent..

[11] Wiriyawattana P, Suwonsichon S, Suwonsichon T (2018). Effects of aging on taste thresholds: A case of Asian people. J. Sens. Stud..

[12] Schiffman SS (1997). Taste and smell losses in normal aging and disease. JAMA.

[13] Shimizu Y (1997). A histomorphometric study of the age-related changes of the human taste buds in circumvallate papillae. Oral Med. Pathol..

[14] Pavlidis P, Gouveris H, Anogeianaki A, Koutsonikolas D, Anogianakis G, Kekes G (2013). Age-related changes in electrogustometry thresholds, tongue tip vascularization, density, and form of the fungiform papillae in humans. Chem. Senses.

[15] Arvidson K (1979). Location and variation in number of taste buds in human fungiform papillae. Eur. J. Oral Sci..

[16] Miller IJ (1989). Variation in human taste bud density as a function of age. Ann. N. Y. Acad. Sci..

[17] Ogawa T (2017). Longitudinal study of factors affecting taste sense decline in old-old individuals. J. Oral Rehabil..

[18] Solemdal K, Møinichen-Berstad C, Mowe M, Hummel T, Sandvik L (2014). Impaired taste and increased mortality in acutely hospitalized older people. Chem. Senses..

[19] Bartoshuk LM, Rifkin B, Marks LE, Bars P (1986). Taste and aging. J. Gerontol..

[20] Murphy C, Gilmore MM (1989). Quality-specific effects of aging on the human taste system. Percept. Psychophys..

[21] Weiffenbach JM, Cowart BJ, Baum BJ (1986). Taste intensity perception in aging. J. Gerontol..

[22] Barragán R (2018). Bitter, sweet, salty, sour and umami taste perception decreases with age: Sex-specific analysis, modulation by genetic variants and taste-preference associations in 18 to 80 year-old subjects. Nutr..

[23] Heft MW, Robinson ME (2014). Age differences in suprathreshold sensory function. Age.

[24] Mojet J, Heidema J, Christ-Hazelhof E (2003). Taste perception with age: Generic or specific losses in supra-threshold intensities of five taste qualities?. Chem. Senses.

[25] Puputti S, Aisala H, Hoppu U, Sandell M (2019). Factors explaining individual differences in taste sensitivity and taste modality recognition among Finnish adults. J. Sens. Stud..

[26] Goto TK, Yeung AWK, Suen JLK, Fong BSK, Ninomiya Y (2015). High resolution time–intensity recording with synchronized solution delivery system for the human dynamic taste perception. J. Neurosci. Methods.

[27] Rocha RAR (2020). Temporal profile of flavor enhancers MAG, MSG, GMP, and IMP, and their ability to enhance salty taste, in different reductions of sodium chloride. J. Food Sci..

[28] Onuma T, Maruyama H, Sakai N (2018). Enhancement of saltiness perception by monosodium glutamate taste and soy sauce odor: A near-infrared spectroscopy study. Chem. Senses.

[29] Gotow N, Moritani A, Hayakawa Y, Akutagawa A, Hashimoto H, Kobayakawa T (2018). Effect of a warm-up sample on stabilizing the performance of untrained panelists in time–intensity evaluation. J. Sens. Stud..

[30] Halpern BP (1986). Constraints imposed on taste physiology by human taste reaction time data. Neurosci. Biobehav. Rev..

[31] Nakamura Y (2012). The temporal change in the cortical activations due to salty and sweet tastes in humans: fMRI and time–intensity sensory evaluation. NeuroReport.

[32] Yamamoto T, Kawamura Y (1981). Gustatory reaction time in human adults. Physiol. Behav..

[33] Yamamoto T, Kawamura Y (1984). Gustatory reaction time to various salt solutions in human adults. Physiol. Behav..

[34] Fozard JL, Vercruyssen M, Reynolds SL, Hancock PA, Quilter RE (1994). Age differences and changes in reaction time: The Baltimore Longitudinal Study of Aging. J. Gerontol..

[35] Der G, Deary IJ (2006). Age and sex differences in reaction time in adulthood: Results from the United Kingdom Health and Lifestyle Survey. Psychol. Aging.

[36] National Institute of Health and Nutrition. Outline of the National Health and Nutrition Survey (NHNS). National Institute of Health and Nutrition. https://www.mhlw.go.jp/bunya/kenkou/kenkou_eiyou_chousa.html (2012–2019).

[37] Schiffman SS (2018). Influence of medications on taste and smell. World J Otorhinolaryngol Head Neck Surg..

[38] Tukey, J. W. *Exploratory Data Analysis*. Vol. 2 (1977).

[39] O'Mahony M, Wong SY (1989). Time-intensity scaling with judges trained to use a calibrated scale: Adaptation, salty and umami tastes. J. Sens. Stud..

[40] Yeung AWK, Wong NSM, Eickhoff SB (2020). Empirical assessment of changing sample-characteristics in task-fMRI over two decades: An example from gustatory and food studies. Human Brain Mapp..

[41] Folstein MF, Folstein SE, McHugh PR (1975). “Mini-mental state”: A practical method for grading the cognitive state of patients for the clinician. J. Psychiatr. Res..

